# Undetected iatrogenic lesions of the anterior femoral shaft during intramedullary nailing: a cadaveric study

**DOI:** 10.1186/1749-799X-3-30

**Published:** 2008-07-17

**Authors:** Stamatios A Papadakis, Charalampos Zalavras, Raffy Mirzayan, Lane Shepherd

**Affiliations:** 1 Department of Orthopaedics, Keck School Of Medicine, LAC+USC Medical Center, University of Southern California, 1200 North State Street, GNH 3900, 9312 Los Angeles, CA 90033, USA

## Abstract

**Background:**

The incidence of undetected radiographically iatrogenic longitudinal splitting in the anterior cortex during intramedullary nailing of the femur has not been well documented.

**Methods:**

Cadaveric study using nine pairs of fresh-frozen femora from adult cadavers. The nine pairs of femora underwent a standardized antegrade intramedullary nailing and the detection of iatrogenic lesions, if any, was performed macroscopically and by radiographic control.

**Results:**

Longitudinal splitting in the anterior cortex was revealed in 5 of 18 cadaver femora macroscopically. Anterior splitting was not detectable in radiographic control.

**Conclusion:**

Longitudinal splitting in the anterior cortex during intramedullary nailing of the femur cannot be detected radiographically.

## Background

Currently the standard treatment for most femoral shaft fractures in adults is intramedullary nailing (IMN) [1], as it offers biomechanical and biologic advantages when compared with other methods of fixation [2]. Although femoral nailing is generally considered a technically demanding procedure, the incidence of iatrogenic complications associated with the technique has not been well documented. Such complications include comminution and, rarely, femoral neck fractures [3].

The purpose of this study is to report the observation of undetected radiographically iatrogenic longitudinal splitting in the anterior cortex during intramedullary nailing of the femur. A review of the English literature revealed no similar reports.

## Methods

As part of an ongoing study on the impact of the localization of the entry point on mechanical complications during nailing, 9 pairs of fresh-frozen intact femora from adult cadavers, stripped of all soft tissues, were studied. Standardized anteroposterior and lateral radiographs were taken in all cadaveric femora, in order to exclude previous fractures or lesions, and to assess the medullary canal diameter.

The nine pairs of femora were separated into 2 groups; one group consisted of right femora and the other of left femora. The method of using pairs of femora from one individual was chosen in order to minimize differences in the anatomical shape and mechanical properties of the two femurs. A standardized antegrade intramedullary nailing technique was used in both groups. Specimens were stored at -20°C. All specimens prior to testing were thawed in a bath of normal saline at 30°C in order to be appropriately hydrated. After they were thawed they were mounted in a holding clamp. The starting point, directly seen, was slightly anterior to the trochanteric fossa at approximately the midline of the femoral neck in the first group (right femora) and at the trochanteric fossa at approximately the junction of the middle and posterior third of the femoral neck in the second group (left femora). A guide wire was placed in the distinct starting point of each group and the proximal femur was opened with a cannulated drill. An end-cutting reamer was used to penetrate the proximal metaphyseal bone. Reaming of 1.5 mm greater than the nail diameter was progressively performed with care to avoid eccentric placement. Distal medullary canal was reamed approximately 3 cm above the intercondylar notch. When 10–13 mm in diameter nails were used, the first 8 cm from the entry portal were reamed to 14 mm in diameter in order to accept the larger diameter proximal end of the nails, according to manufacturer's surgical technique. A fully slotted titanium intramedullary nail with a 230 cm radius of curvature was inserted (Uniflex, Biomet, Inc. Warsaw, IN). Table 1 shows the size of reamers and the diameter of the nails used in the nine pairs of femora. The targeting device was used for the insertion of the proximal locking screw, whereas the distal one was inserted with a free-hand technique under image intensification.

**Table 1:** Size of reamers and diameters of nails

**Pairs**	**Femora**	**Reamer**	**Size of nail**
1	Right*/Left	13.5	12
2	Right/Left	14.5	13
3	Right/Left	14.5	13
4	Right/Left	15.5	14
5	Right*/Left	15.5	14
6	Right*/Left	16.5	15
7	Right/Left	11.5	10
8	Right*/Left	15.5	14
9	Right*/Left	13.5	12

The size of reamers and the diameter of the nails used in the nine pairs of femora. Specimens in the first group (right femora) with anterior femoral splitting are marked with an asterisk.

After the procedure each femur was stored at -20°C to allow an independent observer to perform a complete inspection of every femur on a separate occasion, immediately after they were thawed at room temperature. Every iatrogenic lesion was recorded. Measurements on the femora were performed using a digital caliper (Digimatic, Mitutoyo Corp., Japan, instrumental error ± 0.02 mm). The measurements were performed in random order to avoid bias and the left and right femora of the same pair were never measured sequentially. Four measurements were performed in every femur. These included: a) the distance between the most posterior and anterior margins of the neck in the sagittal plane; b) the distance between the posterior tip of the nail and the posterior margin of the neck. Half of the diameter of the proximal part of the inserted nail was added to this measurement to find the distance of the exact entry point of the nail from the posterior neck margin (absolute location); Subsequently, this distance was divided by measurement (a) to calculate the relative location of the entry point as a ratio of the anteroposterior neck diameter. This would take into account potential differences in dimensions between the study femora; c) the length of the fracture line, if any; d) the distance of the most proximal tip of the split from the neck of the femur. All measurements were made in millimeters (mm).

Standardized anteroposterior and lateral radiographs were taken in all cadaver femora for identification of possible fractures, if any, after the procedures. Moreover, in the presence of a fractured femur a fluoroscopic control was undertaken for the possible detection of the fracture line in different projections.

The absolute and relative location of the entry point in the two groups was compared with the paired t-test. The prevalence of iatrogenic fractures in the two groups was compared using the Fisher's exact test and the relative location of the fractured and intact groups with the two-sample t-test. All tests were two-tailed and p values less than 0.05 were considered significant.

## Results

**Figure 1:**
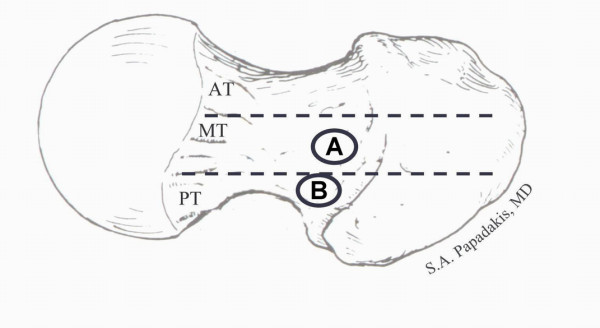
**Point (A) demonstrates the distinct entry point for the first group (right femora), whereas point (B), demonstrates the distinct entry point for the second group (left femora)**. (AT), indicates the anterior third of the femoral neck in the sagittal plane; (MT), the medial third; and (PT), the posterior third.

The average neck-shaft angle of the femora was 131.1 degrees (± 3.4° SD). Only one cadaver femur had an angle of 140 degrees. The average neck-shaft angle for the first group was 132.4 degrees (± 3.5° SD) whereas the average neck-shaft angle for the second group was 130.5 degrees (± 3.3° SD). This difference was not statistically significant. The average distance between the most posterior and anterior margins of the neck in the sagittal plane was 30.5 mm (Figure 1). In the first group the average distance was 31.5 mm and in the second group 29.5 mm. Also, this difference was not statistically significant. Therefore the anatomy of the studied femora did not differ between the two groups.

The average distance of the entry point from the most posterior margin of the femoral neck was 15.4 mm (± 2.3 SD) for the first group and 9.1 mm (± 1.8 SD) for the second group. This difference was statistically significant (p < 0.001, paired t-test). The mean relative position of the entry point expressed as a percentage of the anteroposterior diameter of the superior surface of the femoral neck was 49% (ranging from 41% to 57%) and 31% (ranging from 26% to 36%) for the first and second group, respectively. This difference was also statistically significant (p < 0.001, paired t-test) demonstrating that a distinct entry point selection was achieved in each group (Figure 1). Therefore, the two groups differed only with regards to the exact location of the entry point.

**Figure 2:**
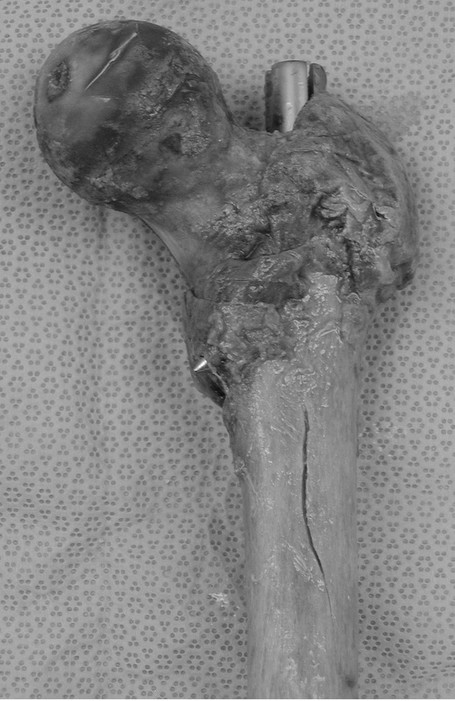
It is evident the longitudinal splitting in the anterior cortex of a femur.

**Figure 3:**
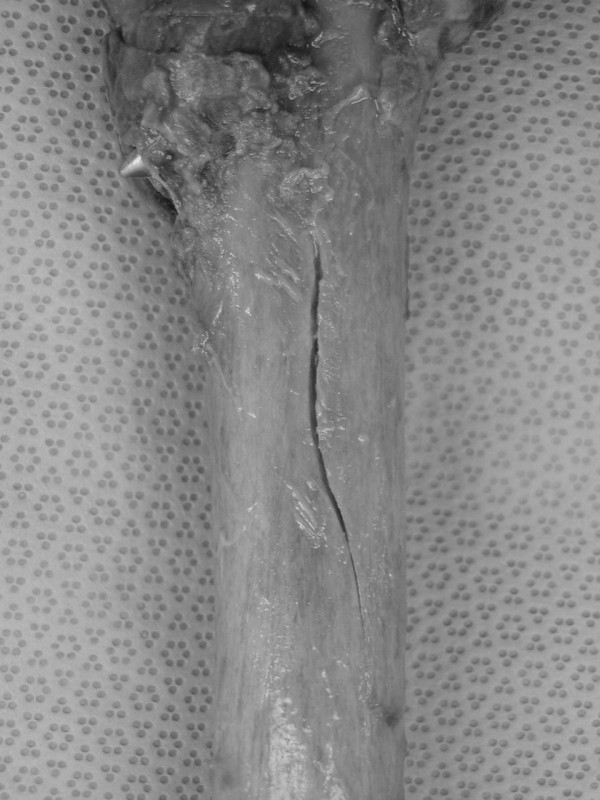
Close-up photo of the splitting in the same femur.

Longitudinal splitting in the anterior cortex was revealed in five of nine cadaver femora (56%) in the first group (right femora) (Figures 2, 3), whereas in the second group (left femora) no splitting was detected. All cases of longitudinal splitting were nondisplaced cracks and there were no cases of bursting of the femur. The increased prevalence of splitting in the first group was statistically significant (p = 0.029, Fisher's exact test). The average length of the longitudinal split was 30.7 mm (± 19.9 SD) and the average width of the split was 2.5 mm (± 1.5 SD). The average distance of the most proximal tip of the split from the neck of the femur was 59.7 mm (± 8.7 SD) and it was at the level of the lesser trochanter in all five fractured specimens. Comparison of the fractured versus the intact specimens showed a significantly more anteriorly placed entry point in the first group; the mean relative position of the entry point was 48% (± 6% SD) in the fractured group versus 37% (± 10% SD) in the intact group (p = 0.04, two-sample t-test).

**Figure 4:**
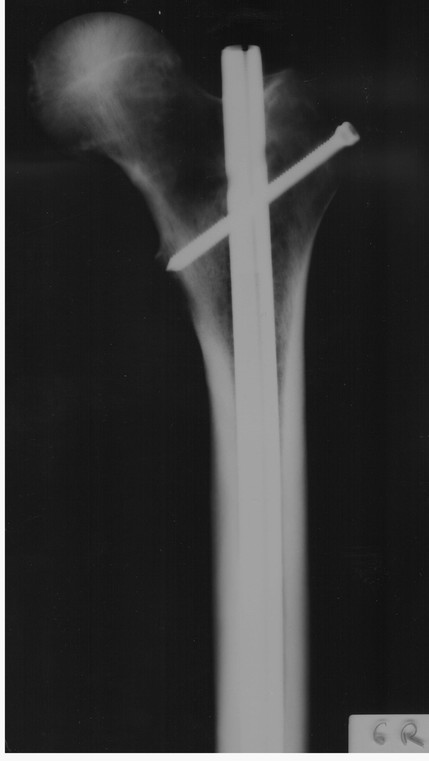
**Anteroposterior radiograph of the same femur**. No evidence of fracture line can be documented.

**Figure 5:**
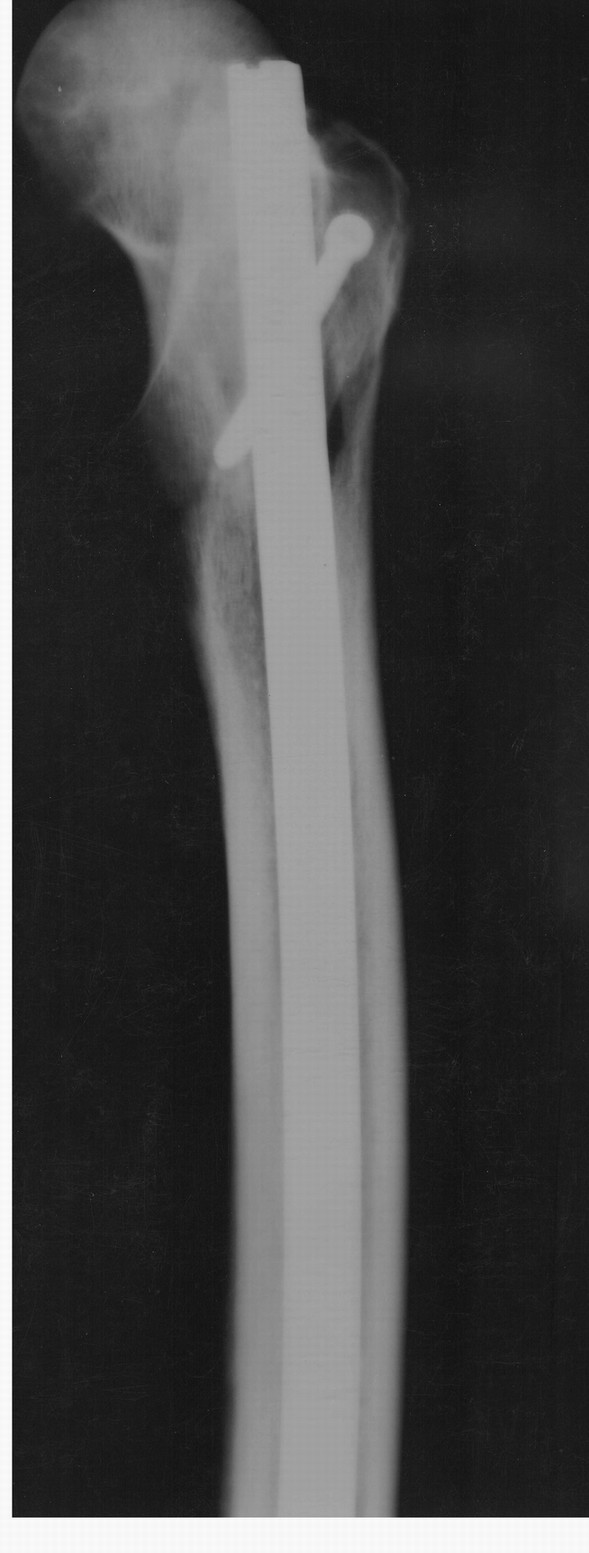
**Lateral radiograph of the same femur**. No evidence of fracture line can be documented.

No other fractures were found during inspection or radiographic control either in the first or in the second group. The radiographic and fluoroscopic control in the five fractured femora revealed no sign of the longitudinal split in anteroposterior and lateral projections (Figures 4, 5).

## Discussion

Treatment of fractures of the femoral shaft can be associated with technical errors leading to numerous iatrogenic complications during closed intramedullary nailing of the femoral shaft [4,5]. An inappropriate entry point for nail insertion into the proximal femur could result in fracture site comminution, proximal femur and even femoral neck fracture [4,6].

Johnson et al [5] found that an entry point too anteriorly to the midline of the femur results in greater hoop stresses in the femoral cortex, and probably in femoral bursting. A biomechanical study carried out by Tencer et al [7] has shown that placement of the starting hole 6 mm or more anterior to the neutral axis of the femur is likely to result in consistent cracking of the proximal femur. Alho et al [8] also reported a 26% prevalence of proximal fragment comminution with an anterior insertion of the intramedullary nail. However, none of these studies used radiographic control to investigate the diagnosis of such lesions.

The placement of the nail more anteriorly to the posterior third of the neck in this study was complicated with a prevalence of 56% of anterior femoral splitting. All of these lesions were seen during nail insertion, at its final phase. A possible explanation could be that during reaming the flexible shaft of the reamers could easily follow the curved path of the medullary canal, as the femur is convex anteriorly. On the other hand, during nail insertion the relevant stiffness of the nail, the mismatch in curvature between the inserted nail and the medullary canal, in combination with an entry point anterior to the trochanteric fossa has possibly led to the femoral splitting. As described in an article by Egol et al [9], the average femoral anterior radius of curvature is 120 cm (SD ± 36) and there is a significant mismatch between the anatomical radius of curvature of the femur and most intramedullary nails, including the one used in this study. In the same study, the analysis of the radii of curvature of 8 current antegrade intramedullary nails demonstrated that they all have a greater radius of curvature ranging from 186 to 300 cm (straighter) than that of the average femur. However, this is only one factor affecting nail insertion. As previously stated the placement of the starting point slightly anteriorly to the neutral axis of the medullary canal forces the nail to travel anteriorly and thus, increased hoop-stresses cause splitting of the anterior femoral cortex [5,7].

C-arm images or plain films were used to evaluate the specimens after fixation, as this type of radiographic analysis is commonly used in clinical practice. C-arm fluoroscopy is the preferable means of monitoring IMN intraoperatively. However, C-arm images often do not reveal subtle fractures that plain radiographs might, as the quality of the images using C-arm fluoroscopy is usually worse than that of plain radiographs [10].

In this study, the anterior splitting was not detectable either in radiographic or fluoroscopic examination apparently because of the vertical orientation of the fractures and the overlapping density of the bone cortex and the intramedullary nail. Imaging confirmation of the splitting by using either radiographs obtained in multiple different projections other than the anteroposterior and lateral ones, or a CT-scan study, was not performed as these means are not used intraoperatively.

The bone density of the cadaveric femora was not tested. By using pairs of intact femurs from one individual donor, any variability in the mechanical properties of the paired specimens is minimized and differences in the bone strength are not expected. Moreover, there were no simulated diaphyseal fractures, as it is generally difficult to simulate identical patterns of fractures (i.e., single, comminuted, etc.).

It should be noted that this study has several limitations. The main weakness is that this study involved intramedullary nailing of intact cadaveric femora; therefore our findings may not be replicated in the surgical practice of intramedullary nailing of femur fractures for two reasons. First, cadaveric bone stripped of soft tissue, frozen, and thawed is hard and has very limited ability of compliance compared to live bone; therefore the effects of the location of the entry point and of the curvature mismatch between the nail and the medullary canal were magnified. Second, intact femora were nailed, which again augmented stresses on the femur; in the presence of a fracture, entry point variations and curvature mismatch may be accommodated to a degree by displacement at the fracture site. However, this study used matched pairs of femurs, the identical implant by a single manufacturer, and a standardized antegrade nailing technique with the exception of the entry point; all cases of anterior splitting occurred in femora with more anteriorly located entry points, which emphasizes the importance of the location of the entry point in the worst case scenario of a hard and incompliant intact femur.

Finally it should be noted that all cases of longitudinal splitting were nondisplaced cracks undetectable fluoroscopically or radiographically. Hence, it could be argued that such a nondisplaced crack may have minimal effect on the stability of fixation and, consequently, minimal clinical relevance. However, this necessitates static locking of the nail to impart rotational and longitudinal stability to the construct [2]. Although anterior femoral splitting has never been described, detected or led to clinical relevant complications as seen by non-existing literature, we feel that surgeons should be aware of this potential complication, especially if the entry point used is too anterior. Our findings add support to the current recommendation of static IMN for all types of femur fractures [11].

In conclusion, this study has drawn attention to the risk of undetectable iatrogenic splitting in the anterior cortex during antegrade intramedullary nailing of intact cadaveric femora. The clinical significance, if any, of this lesion is unknown.

## Competing interests

The authors declare that they have no competing interests.

## Authors' contributions

SAP, CZ, RM, and LS participated in the design of the study, analysis and writing of this manuscript. SAP and CZ participated also in revising critically the manuscript. All authors read and approved the final manuscript.
